# Morphological characterization of the hippocampus: a first database in Ecuador

**DOI:** 10.3389/fnhum.2024.1387212

**Published:** 2024-10-18

**Authors:** Stefano Buitrón Cevallos, Alex X. Jerves, Clayreth Vinueza, Dennis Hernandez, Carlos Ávila, Andrés Auquilla, Óscar Alvear

**Affiliations:** ^1^Fundación INSPIRE, INSπRE, Quito, Ecuador; ^2^Unidad Académica de Informática, Ciencias de la Computación, e Innovación Tecnológica, Universidad Católica de Cuenca, Cuenca, Ecuador; ^3^Facultad de Medicina, Universidad Internacional del Ecuador, Quito, Ecuador; ^4^Centro Radiológico, Medimágenes, Quito, Ecuador; ^5^Universidad UTE, Facultad de Ciencias, Ingeniería y Construcción, Carrera de Ingeniería Civil, Quito, Ecuador; ^6^Department of Computer Science, University of Cuenca, Cuenca, Ecuador

**Keywords:** Andean patients, database, hippocampus, level sets, MRI, morphological parameters, volume

## Abstract

**Introduction:**

The hippocampal volume is a well-known biomarker to detect and diagnose neurological, psychiatric, and psychological diseases. However, other morphological descriptors are not analyzed. Furthermore, not available databases, or studies, were found with information related to the hippocampal morphology from Latin-American patients living in the Andean highlands.

**Methods:**

The hippocampus is manually segmented by two medical imaging specialists on normal brain magnetic resonance images. Then, its morphological qualitative and quantitative descriptors (volume, sphericity, roundness, diameter, volume-surface ratio, and aspect ratio) are computed via 3D digital level-set-based mathematical representation. Furthermore, other morphological descriptors and their possible correlation with the hippocampal volume is analyzed.

**Results:**

We introduce a first database with the hippocampus’ morphological characterization of 63 patients from Quito, Ecuador, male and female, aged between 18 and 95 years old.

**Discussion:**

This study provides new research opportunities to neurologists, psychologists, and psychiatrists, to further understand the hippocampal morphology of Andean and Latin American patients.

## 1 Introduction

The human’s hippocampus, given its name by Giulio Cesare Aranzio in the sixteenth century due to its similarity in shape with a seahorse, is a brain structure located in the temporal lobe, commonly associated with the episodic and spatial memory, creation of new memories, and the linking between emotions and memories. In the last decades, it has been found that changes in the morphology of the hippocampus, in specific, its volume, are correlated with neurological, psychiatric and psychological diseases such as Alzheimer, temporal lobe epilepsy, rabies encephalitis, global cerebral ischemia, Korsakoff syndrome, senile dementia and Attention Deficit Hyperactivity Disorder (ADHD) (Fernández-Ruiz et al., 2019; Sone et al., 2016; Torres, 2023). Considering the hippocampal volume as a known biomarker to detect and diagnose these diseases (Chaves et al., 2018).

Thus, given the high importance of the hippocampal volume as a biomarker, several studies have been performed to characterize it, establishing statistical information from healthy and sick patients (McHugh et al., 2007; Nobis et al., 2019; da Silva Filho et al., 2017). The aforementioned statistics have been analysed in relation to age and sex. For those studies the hippocampal volume has been obtained by manual (Rogers et al., 2012) and automatic (Hardcastle et al., 2020; Ystad et al., 2009) segmentation techniques applied to Magnetic Resonance Images (MRI) of the brain.

Furthermore, there are no reported studies that relate other hippocampal morphological parameters (e.g., roundness, sphericity, diameter, aspect ratio, and volume-surface ratio) to neurological, psychiatric and psychological diseases, where they could potentially be used to detect, diagnose and prevent them.

In the same way, and despite the importance of hippocampal atrophy measurement for mental health diagnosis, the technology to calculate the hippocampal volume from normal head MRIs is not available in most countries from Latin America.

Available databases in which studies on the hippocampus are stored [e.g., The Alzheimer’s Disease Neuroimaging Initiative (ADNI)] (Petersen et al., 2010) do not include MRIs of patients from Latin American countries. Furthermore, not available databases, or studies, were found with information related to the morphological characterization of the hippocampus from Andean patients living in high altitude.

Giving rise to a fundamental question: *Are the hippocampal volumes reported by studies from other countries similar to their counterparts from Quito, Ecuador?* Furthermore, considering the geographical, cultural, ethnical, and dietary differences among countries, this study establishes new research opportunities to determine *if the morphology of the hippocampus, particularly its volume, is affected by one or more of these factors.*

**Figure 1:**
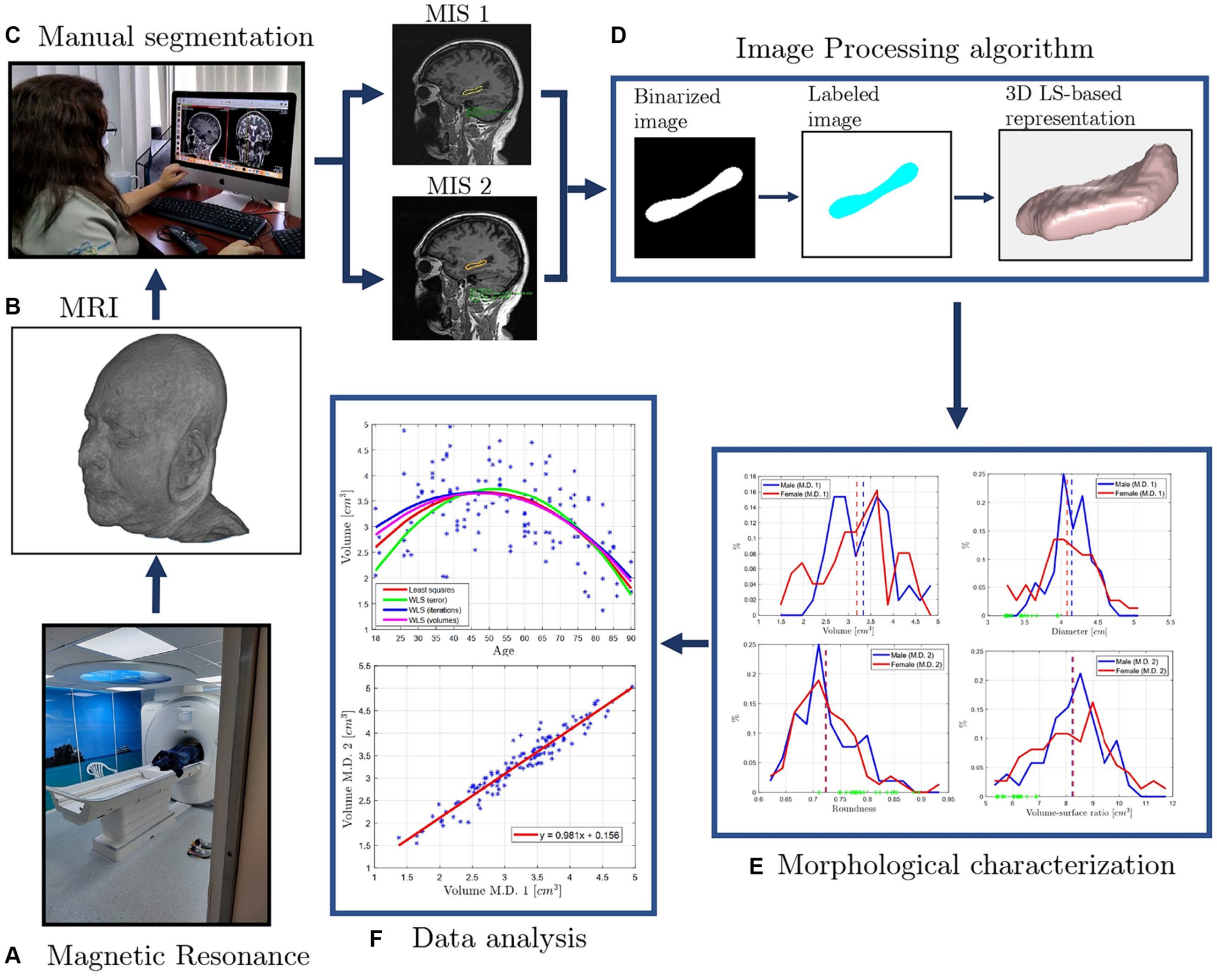
Morphological characterization and analysis process of Ecuadorian patients’ hippocampi mor-phological descriptors from manually segmented normal brain magnetic resonance images (MRI): first, **(A)** a magnetic resonator scanner is used to **(B)** obtain a MRI consisting of volumetric sequences. The present work uses MRIs from the database of the imaging center “Medimagenes” located in Quito, Ecuador. **(C)** Then, the coronal and sagittal views of each MRI are visually inspected by two Medical Imaging Specialists (MIS), finding the slices where the hippocampi (left and right) are located. Then, each MIS (named MIS 1 and MIS 2, respectively) manually trace the hippocampus on each slice from the sagittal view. **(D)** The images are filtered through an image processing algorithm, using the traces as masks to segment the hippocampus, creating accurate shape hippocampus digital twins via the 3D level set-based mathematical functions. Further, computational geometry techniques and algorithms are used to compute **(E)** morphological descriptors of the hippocampus, such as volume, roundness, diameter, aspect ratio, and volume to surface ratio. Finally, **(F)** an analysis of the results is performed, comparing the data obtained for each trace of the medical imaging specialists, and determining possible correlations among volume, age, sex, and the other morphological parameters.

As an attempt to answer the first fundamental question, a procedure to calculate the morphological descriptors of the hippocampus from manually traced normal brain MRI of patients from Quito, Ecuador, aged 18–95 years, is introduced by this work, and depicted in Figure 1. Hence, at the heart of this work lies the *creation of the first database in Ecuador, containing not only hippocampal volumes, but also other morphological descriptors, as well as the manually segmented images from Andean patients living in Quito-Ecuador.* Finally, the obtained hippocampal volumes are compared to results reported by studies from around the globe.

The present work is divided in two sections. First detailing the applied materials and methods, including the participants, MRI protocol, manual segmentation methodology (see Figures 1B,C), image processing algorithm to obtain accurate 3D level-set-based digital representations of the hippocampus, morphological parameters calculation, and the statistical methods applied for data comparison. The second section reports the results of the study, analyzing and classifying the hippocampal volume of the patients from Quito, Ecuador. The classification is performed by sex and the brain’s hemisphere, so the results can be compared to those reported by other studies (Cook et al., 1992; De Francesco et al., 2021; Mangesius et al., 2022; Mu et al., 2020; Özdemir et al., 2019; Viña-González et al., 2021; Ystad et al., 2009). Furthermore, the analysis of other morphological parameters of the hippocampus (e.g., sphericity, roundness, diameter, aspect ratio, and volume to surface ratio, see Figures 1E,F) is introduced in a first attempt to find new correlations for more accurate predictors as well as biomarkers.

## 2 Materials and methods

### 2.1 Study population

The MRI’s data was selected from the medical imaging center Medimagenes’ repository in Quito, Ecuador, specifically, from patients that had an order by their medical specialist due to related symptoms. Furthermore, the data was limited to a pre-pandemic period (2019–2020). Based on this information, a significant sample of patients from Quito that may require a simple brain MRI is calculated with Equation 1:


(1) $$n=\frac{N*Z^{2}*p*q}{e^{2}*\left(N-1\right)+Z^{2}*p*q}$$

where n is the sample size, N is the population’s size (patients from Quito), Z is a statistic parameter that depends on the confidence level, e is the maximum accepted estimated error, p is the success probability, and q is the failure probability. Considering that the population of Quito in the pre-pandemic period was of approximately 2′781.641 (INEC, 2017), and subtracting people under 18 years old (27%) and considered under poverty and extreme poverty (11%), respectively (Quito Como Vamos, 2020), a N value of 1′724.617 is used. To determine the probability *p*, the number of people who have had a simple brain MRI in the United States was considered. Hence, it is reported that 11% of the population had an MRI in 2017 (Statista, 2024), and assuming that 1/3 of those images corresponded to a simple brain study, the probability *p* would be of 3.5%, and therefore, the *q* probability value of 96.5%. Applying a confidence level of 95% (corresponding to a Z value of 1.90), and an estimated error e of 5%, the sample size is obtained:


$$n=\frac{1724617*1.90^{2}*0.035*0.965}{0.05^{2}*\left(1724617-1\right)+1.90^{2}*0.035*0.965}\approx 49$$

Available data for the study consists of normal brain MRIs from 63 patients from Quito, Ecuador, 26 male and 37 female, aged 18–95 years (54.62 ± 18.65), which is greater to the calculated sample size.

The participants’ geographic location corresponds to an altitude that ranges from 2,500 to 3,000 meters above the sea level in the Andes Mountain chain, and a latitude of 0.22985°. In general, the Ecuadorian population mainly consists of mestizos, and their diet varies depending on their location. For example, a study detected high sodium consumption in the Coastal region (Sánchez-Llaguno et al., 2013), while the diet in the Sierra region (the Andean mountains) is based on carbohydrates, mainly potato, followed by rice, noodles, oat and sugars (Calle, 2022; FAO, 2017; Oyarzun et al., 2013).

The participants involved in this study signed an informed consent in order to use their images, specifying that their personal information was going to be anonymized, assigning, instead, an id number to each patient. Moreover, information, such as age or sex, is later used for data analysis and statistics.

### 2.2 MRI protocol and segmentation method

The MRIs consist in T1 weighted volumetric sequences [Repetition Time (TR) = 2,200 ms, Time to Echo (TE) = 2.95 ms, matrix = 256×256 pixels, resolution = 1 mm/pixel, slice thickness = 1 mm], acquired with a 3 Tesla Siemens MAGNETOM Spectra scanner.

Segmentation of the hippocampus can be performed by several methods such as automatic (e.g., atlas-based segmentation, Machine Learning approach), semi-automatic, and manual. However, the first two methodologies tend to overestimate the hippocampal volume. Thus, manual segmentation is still considered as the gold standard to characterize the hippocampus (Barragán-Campos et al., 2015). Furthermore, most automatic segmentation software (e.g., FreeSurfer) use algorithms built with available data in which Andean patients living in high altitudes are not included. Hence, these type of software have not been included for this study in order to avoid bias in the results.

All the MRIs were visually segmented by two independent Medical Imaging Specialists (MIS), who rigorously followed the Joint EADC-ADNI (European Alzheimer’s Disease Consortium-Alzheimer’s Disease Neuroimaging Initiative) Harmonized protocol (Boccardi et al., 2011), whose main characteristics are summarized for completeness as follows:

- The studies, stored in a DICOM format, are loaded in the HOROS Imaging software (HOROS Project, 2018), were three different views (axial, coronal, and sagittal) of the normal brain MRIs are available.
- The coronal and sagittal views are compared, selecting the slices where the hippocampi (left and right) are located. The main hippocampal regions considered were the head, body, and tail. It is worth mentioning that more than one view is required to properly identify the voxel’s gray-scale intensities that correspond to the hippocampal region.
- Then, the selected slices for the left and right hippocampi, respectively, are extracted from the sagittal view.
- With the help of a ROI (region of interest) tool of the HOROS Imaging software, the hippocampus is manually traced slice by slice, following a rostrocaudal direction. It is important to exclude some parts surrounding the hippocampal region, such as the amygdala, choroid plexus, and fornix. On the other hand, other parts need to be included in the segmentation, i.e., the alveus and fimbria.
- Each MIS traces the entire set of hippocampi in an independent way, so bias is avoided and corrected. The process ends saving the traced image in DICOM format, keeping the original MRI and the traced one.

**Figure 2:**
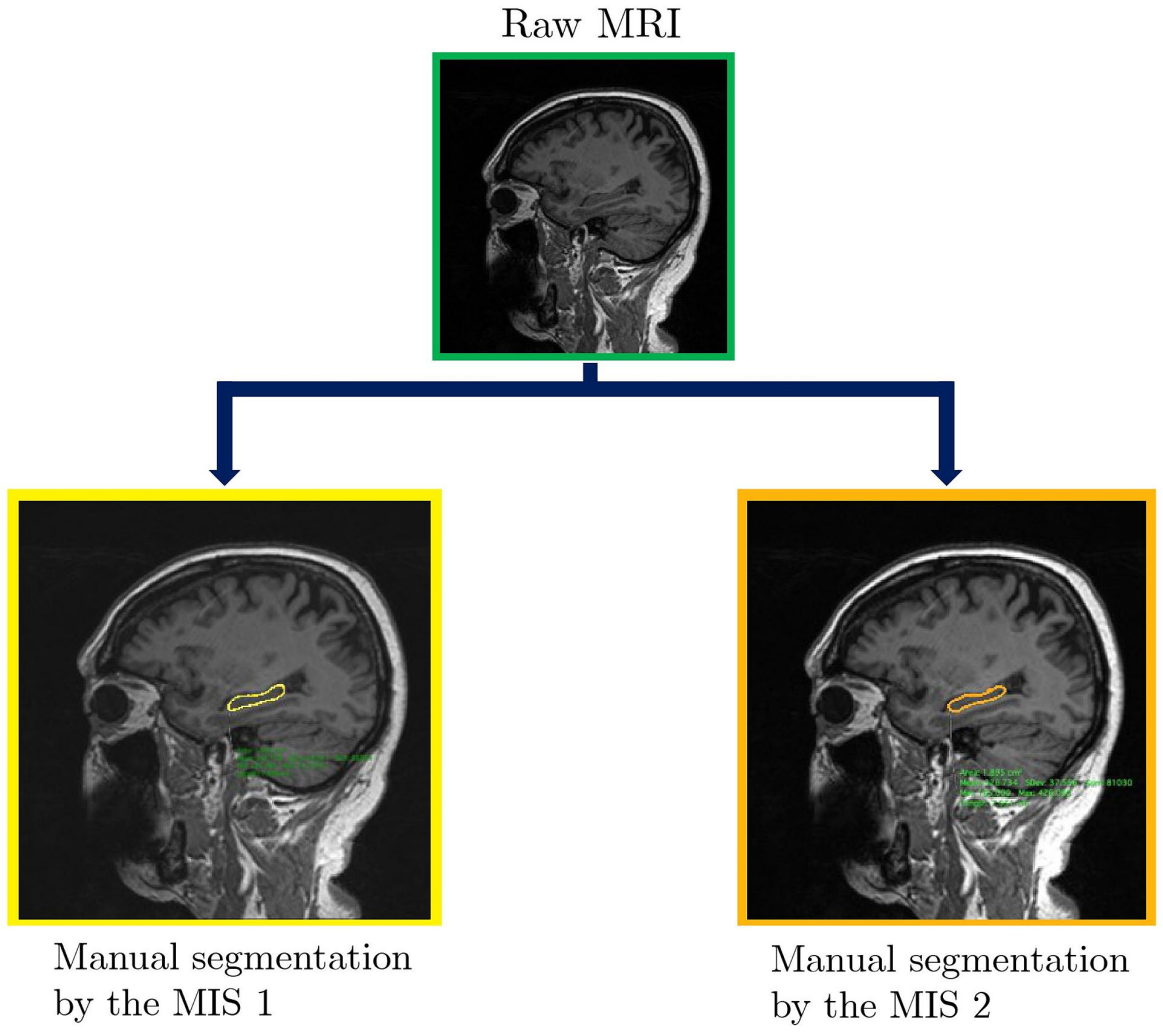
Comparison between the manual segmentation of the hippocampus performed by two MIS.

A first open access database HipoML (2023) is created with the raw and manually traced images obtained after the tuning phase was completed (see Figure 2). The images from the database are used for this study.

### 2.3 Image processing and morphological parameters calculation

3D level set (LS) functions have proven to effectively represent complex natural shapes extracted from different imaging techniques [e.g., X-ray computed tomographic images (XRCT), magnetic resonance images (MRI)]. Level set functions allow to digitally and mathematically represent an object in 3D, so computational geometry algorithms can then be applied to quantitatively and qualitatively calculate morphological descriptors such as volume, surface area, roundness, sphericity, aspect ratio, volume to surface ratio. The definition of the aforementioned descriptors are taken from (Cho et al., 2006; Jerves et al., 2016; Medina and Jerves, 2019). For instance, sphericity is defined in Equation 2:


(2) $$S=\frac{r_{in,\max}}{r_{cir,\min}}$$

Where $r_{in,\max}$ is the maximum inscribable radius and $r_{cir,\min}$ is the minimum circumscribable radius of the hippocampus. Roundness is defined in Equation 3:


(3) $$R=\frac{\frac{1}{N}\;\sum_{i=1}^{N}r_{i}}{r_{cir,\min}}$$

where $r_{i}$ is the radius of curvature at the ith corner and N is the total number of corners. Aspect ratio is defined in Equation 4:


(4) $$AR=\frac{\min_{prin.-dir.}}{\max_{prin.-dir.}}$$

Where $\min_{prin.-dir.}$ is the minimum principal direction and $\max_{prin.-dir.}$ is the maximum principal direction of the hippocampus.

Traditional image processing algorithms may be challenging to apply to MRIs, mainly depending on the tissue of interest to be segmented. Specifically, the segmentation of the hippocampus is difficult, since the pixel intensity values of the brain’s MRI are similar to each other (Fischl et al., 2002), having to discard well known techniques such as thresholding. Hence, other techniques have to be considered (Dill et al., 2014; Jalab and Hasan, 2019; Uhl et al., 2018), including the atlas-based segmentation (Carmichael et al., 2010; Pipitone et al., 2014) or a machine learning approach (Baldeón, 2020; Carmo et al., 2021) with a process known as semantic segmentation.

**Figure 3:**
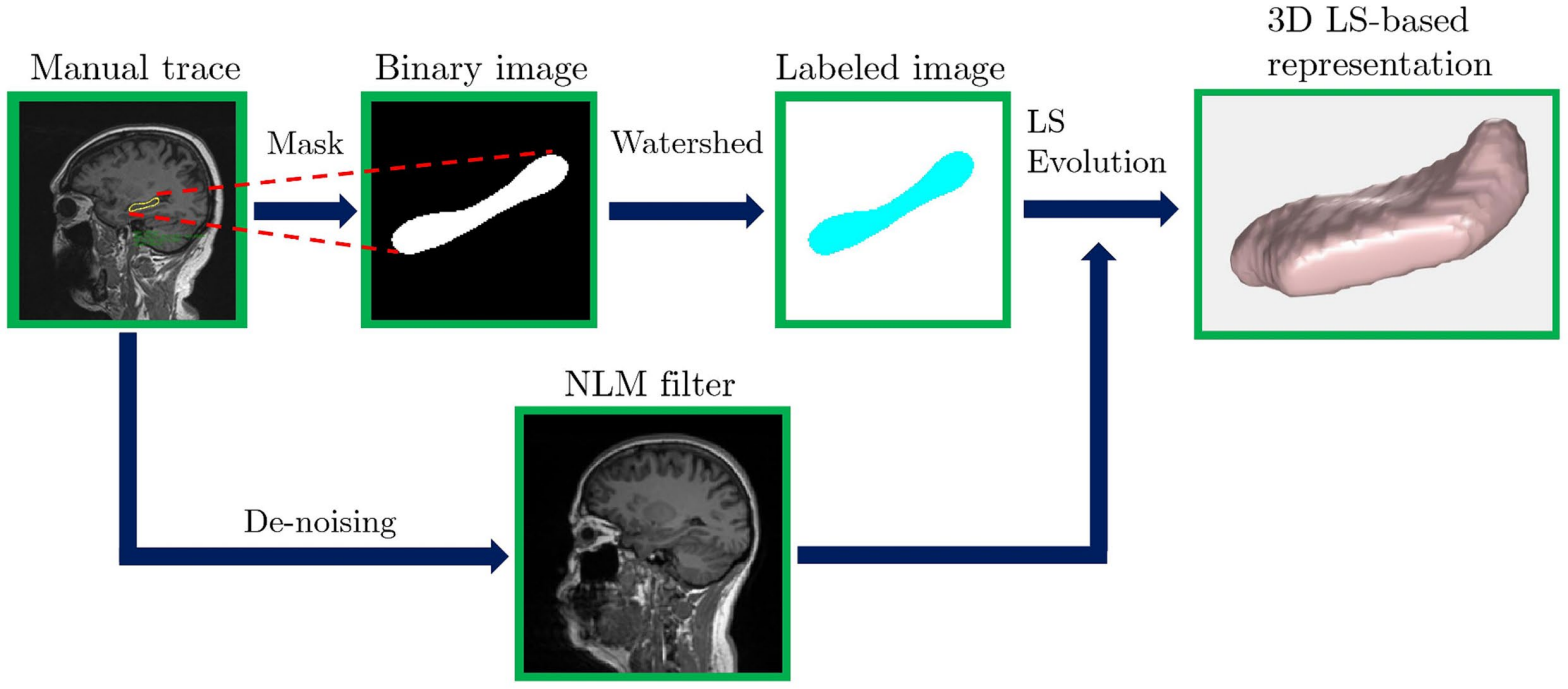
Image processing algorithm used to obtain a 3D level set-based digital representation of the hippocampus from a manually traced brain MRI.

In this work, the hippocampus is digitally represented via 3D-level-set mathematical functions, following the process illustrated in Figure 3 and described next. First, the hippocampus is manually segmented with traces performed by two MIS, the traces are used as masks, where the pixels inside the trace are assigned a value of 1, and the remaining pixels in the image are excluded (or turned to a value of 0), obtaining a binarized image. Second, a watershed algorithm (Meyer, 1994) is applied to the binary image, labeling the pixels that correspond to the hippocampus. Third, the level set evolution algorithm (Kawamoto et al., 2016; Vlahinić et al., 2014) uses as input the previously described labeled image and a de-noised MRI (Buitrón Cevallos et al., 2023), that comes as the output of a non-local means (NLM) filter. Finally, the hippocampus geometry is fully, accurately, digitally, and mathematically captured by a 3D level-set function.

The image processing algorithm, described in Figure 3, is applied to the MRIs of each participant, obtaining an accurate digitized 3D representation of each hippocampus. Then, the digital representation is used to compute the aforementioned morphological descriptors. The data is then analyzed to find possible correlations among the morphological parameters, specifically, correlations with the hippocampal volume, since it is a broadly known biomarker for detection and diagnosis of neurological, psychiatric, and psychological diseases.

### 2.4 Statistical analysis

Given the amount of available data (126 measurements in total), the data normality was determined using the Kolmogorov–Smirnov test (*p* > 0.05), comparing the cumulative distribution function (CDF) between the volume of the hippocampi with that of a normal distribution. Then, a two-tailed *t*-test (*p* > 0.05) was applied to compare the volume of both hippocampi (left and right) and also determine statistically significant differences regarding the patients’ sex.

## 3 Results

### 3.1 Hippocampal volume analysis

As previously mentioned in subsection 2.3, hippocampus’s morphological descriptors can be computed directly from its 3D-level-set mathematical representation. In this section, the hippocampal volume of 63 Ecuadorian patients is characterized, and then compared to the results from studies around the globe. We believe that variables such as altitude, latitude, culture, diet, climate, might play a role in the morphology of the hippocampus. Thus, some changes may be found depending on the country where the patients live in.

**Figure 4:**
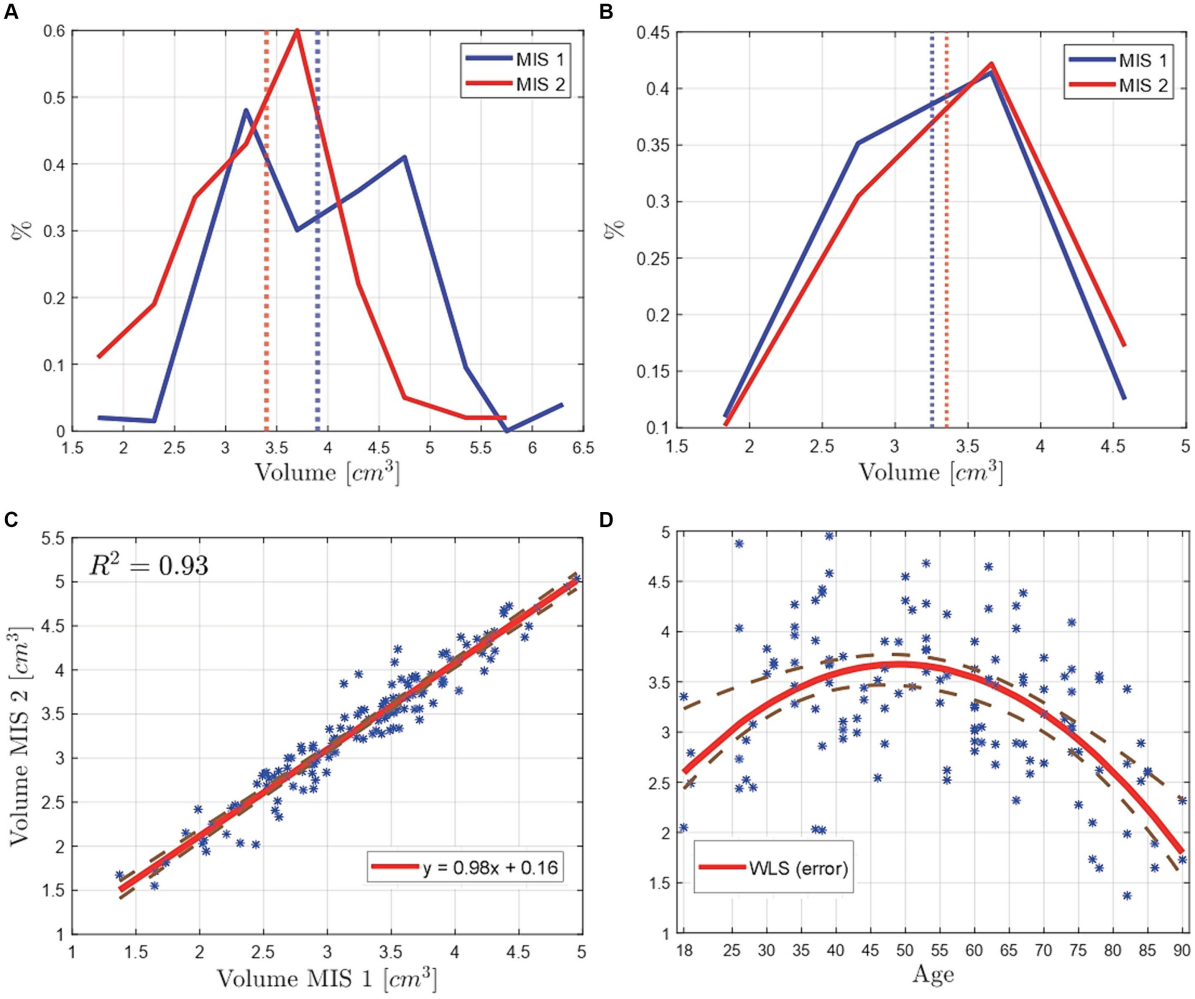
Volume comparison of the manually segmented hippocampi of 63 Ecuadorian patients by two MIS. The vertical line represents the PDFs mean value of the data. **(A)** Baseline, where the volume’s mean difference is of 0.5 cm^3^. **(B)** Results from the tuning phase, where the volume’s mean difference is of 0.1 cm^3^. **(C)** Scatter plot of the calculated hippocampal volumes from the manual segmentation of the MIS 1 vs. the manual segmentation of the MIS 2. The data was correlated with a linear regression model, reporting a *R*^2^ of 0.93. **(D)** Scatter plot of the hippocampus’ volume vs. age. The data was fitted by means of a weighted least squares (WLS) method, where the weights correspond to the difference between the volume data points and the least squares’ fitted data points.

In order to analyze the hippocampal volume, its distribution is plotted (see Figure 4), displaying with a blue line the data resulting from the tracing process of MIS 1, with a red line the data from MIS 2, and with dotted vertical lines their mean values. In Figure 4A, a difference is found between the two MIS, with mean values of 3.4 and 3.9 cm^3^, representing a difference of 0.5 cm^3^, which corresponds to an approximate mismatch of 13%.

**Figure 5:**
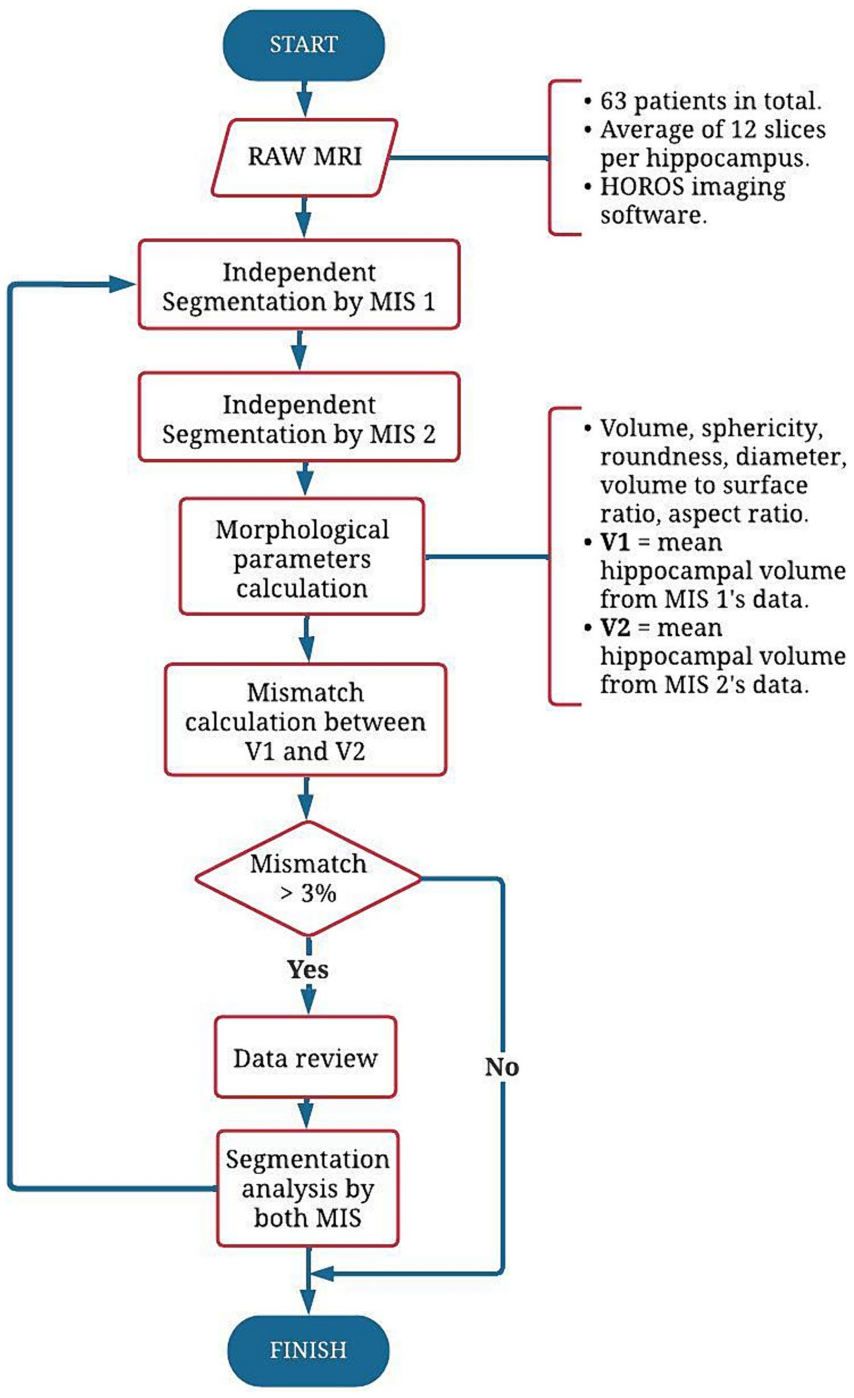
Flow chart describing the tuning phase in the manual segmentation procedure. First, an independent segmentation by each MIS is performed on each MRI. Then, the hippocampal morphological descriptors are obtained from its 3D level set-based representation, comparing the results in volume. If the data mismatch is >3%, both MIS analyze the segmentations, and discuss on the obtained data. The process is repeated until the mismatch percentage is lower or equal than 3%.

The error is caused due to the number of hippocampal volumes that did not match between the seg-mentation done by each MIS. When comparing the hippocampal volumes (in cm^3^), a tolerance of ±3% was considered. Out of 126 volumes, just 25 matched, which represents a matching percentage of 20%. These results were used as a baseline to improve the segmentation process, following a tuning phase described in Figure 5. Thus, the information was analyzed by both MIS, repeating the segmentation process until the mismatch percentage was lower than 3%. When the tuning phase was finished, a coincidence of 95% was obtained, displaying the final results in Figure 4B, noting a hippocampal volume from MIS1 of 3.25 cm^3^ and from MIS2 of 3.35 cm^3^, corresponding to a difference of 0.1 cm^3^ between the volumes’ mean values, which represents an approximate mismatch of 3.03%.

The correlation between the hippocampal volume distributions from the two MIS is visualized with the help of a scatter plot (see Figure 4C). The data was fitted implementing a linear regression model, reporting a *R*^2^ of 0.93 which indicates that the data has a strong lineal correlation, i.e., the data from the two MIS on the same patient’s normal brain MRI is very similar. The equation of the linear fitting is 𝑦 = 0.981𝑥 + 0.156, the slope is close to 1 and the volumes from the MIS 2 are higher, in average, by a value of 0.156 cm^3^ than the ones from its counterpart. This suggests that the hippocampal volumes obtained from the manual trace of the MIS 2 tends to be by default larger than the hippocampal volumes obtained from the manual trace of the MIS 1. The percentage error was calculated with Equation 5:


(5) $$E=\frac{b}{\bar{x}}$$

where b is the y-intercept of the linear equation (0.156 cm^3^) and $\bar{x}$ is the mean between the data from MIS 1 and MIS 2 (3.286 cm^3^), yielding an error of 4.73%.

The relation between age and hippocampal volume of Ecuadorian patients is illustrated in Figure 4D. In order to visualize the behavior of the data, a fit by means of least squares and weighted least squares (WLS) techniques with polynomial functions is performed (finding the best fit to be corresponded to quadratic polynomials). The best fit reports a *R*^2^ of 0.46, using as weights the difference between the volume data points and the least squares fitted data points.

#### 3.1.1 Comparison and validation with studies from other countries

For patients from Quito, the plot describes a rising curve until it reaches a maximum volume of 3.7 cm^3^ at age of 55, then it starts decreasing until it reaches the minimum volume of 1.7 cm^3^ at age 90. The aforementioned analysis is similar to the results reported by a study in China (Mu et al., 2020), where a scatter plot of the hippocampal volume vs. age of 198 healthy Chinese participants (aged 6–26 years) was performed. Even if the age gap is smaller, the curve is similar, with an inverse u-shape which raises and then starts decreasing.

**Figure 6:**
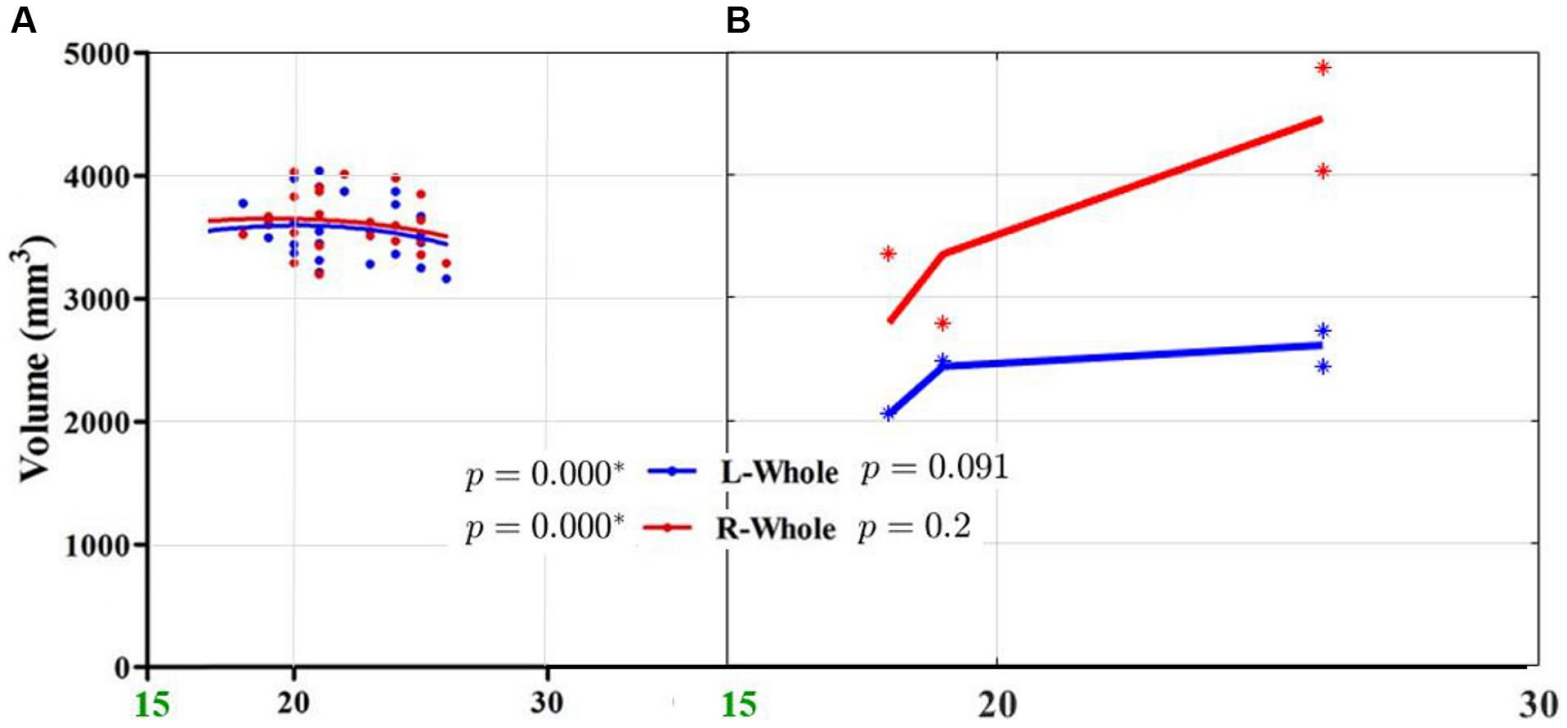
Hippocampal volume vs. age (18 to 26 years old) comparison between: **(A)** right and left hippocampi’s data points from Chinese patients (* indicates a significant relation between age and volume, see the original Figure in Mu et al., 2020), and **(B)** right and left hippocampi’s data points from Ecuadorian patients available at HipoML database (HipoML, 2023), indicating no significant relation between age and volume since *p* >0.05 (it is worth pointing out that just eight data points are available).

In order to compare results from China and Ecuador, data points of Chinese patients under 18 years old were discarded. Furthermore, only the data corresponding to an age gap of 18–30 years old was plotted, as visualized in Figure 6. It is worth noticing that hippocampal volume of Chinese patients’ starts to decrease, unlike the data from Ecuador, where it is still increasing.

Similarly, as with the results from China, we proceeded to compare the hippocampal volume of Ecuadorian patients with that of patients from other countries. An overview of the data is given in Table 1, including the number of participants in the study, their age, and the corresponding mean hippocampal volume.

**Table 1:** Hippocampal volume (cm^3^) comparison with studies from other countries.

(A) Statistical *t*-test comparison							
Country		Number of patients	Age range (mean)	Mean volume	*t-*value	t-crit	*p*-value
Turkey (Ankara)		302 (healthy)	11–84 (45.16)	3.81 ± 0.49	7.11	1.96	<0.001
Italy (Brescia)		64	20–92 (66)	3.74 ± 0.34	6.37	1.99	<0.001
USA (ADNI)		68	56–90 (70)	3.67 ± 0.41	7.19	1.97	<0.001
Norway	Oslo	84	47–75 (65.1)	3.51 ± 0.37	2.80	1.98	0.009
	Bergen	86	46–77 (59.3)	3.66 ± 0.40	5.03	1.97	<0.001
China (Beijing)		198	6–26 (12.27)	3.45	–	–	–
Austria (Innsbruck)		10	44–85 (10.25)	3.44	–	–	–
Ecuador (Quito)		63	18–95 (54.62)	3.30 ± 0.74	–	–	–
UK (London)	Healthy	10	21–36 (30)	3.21 ± 0.40	1.10	2.10	>0.20
	TLE	19	17–56 (32)	3.25 ± 0.81	1.76	2.01	0.062
	FLE	20	18–61 (30)	3.08 ± 0.51	–	–	–
Cuba (Artemisa City)		104	60–87 (13.77)	2.92 ± 0.54	0.65	1.96	>0.20

(B) Comparison by sex and brain’s hemisphere										
Country		RH volume	LH volume	MH volume (#)	FH volume (#)	Ecuador’s patients				
						Patients	RH volume	LH volume	MH volume (#)	FH volume (#)
Turkey		3.86 ± 0.48	3.78 ± 0.49	3.94 ± 0.49 (118)	3.74 ± 0.42 (184)	60	3.53 ± 0.65	3.19 ± 0.73	3.41 ± 0.63 (25)	3.32 ± 0.76 (35)
Cuba		2.96 ± 0.55	2.89 ± 0.53	–	–	29	3.22 ± 0.67	3.01 ± 0.77	–	–
UK	Healthy	3.19 ± 0.39	3.23 ± 0.40	3.15 ± 0.47 (6)	3.29 ± 0.20 (4)	9	3.79 ± 0.69	3.26 ± 0.67	3.58 ± 0.87 (5)	3.52 ± 0.51 (4)
	TLE	3.10 ± 0.75	3.22 ± 0.86	3.58 ± 0.63 (10)	2.98 ± 0.85 (9)	35	3.73 ± 0.59	3.28 ± 0.71	3.58 ± 0.73 (15)	3.45 ± 0.66 (20)
	FLE	3.09 ± 0.52	3.07 ± 0.49	3.29 ± 0.36 (11)	2.81 ± 0.54 (9)	38	3.69 ± 0.57	3.26 ± 0.69	3.55 ± 0.69 (16)	3.42 ± 065 (22)
Norway	Oslo	3.64 ± 0.38	3.38 ± 0.35	3.53 ± 0.39 (24)	3.48 ± 0.33 (60)	31	3.56 ± 0.57	3.35 ± 0.57	3.35 ± 0.46 (13)	3.53 ± 0.64 (18)
	Bergen	3.79 ± 0.40	3.55 ± 0.39	3.72 ± 0.44 (26)	3.61 ± 0.35 (60)	33	3.52 ± 0.60	3.28 ± 0.63	3.35 ± 0.46 (13)	3.43 ± 0.71 (20)
USA (ADNI)		3.73 ± 0.41	3.61 ± 0.40	–	–	32	3.19 ± 0.72	2.98 ± 0.75	–	–
Italy		3.74 ± 0.34	3.64 ± 0.33	–	–	61	3.47 ± 0.71	3.16 ± 0.74	–	–

From Table 1, an overview of the hippocampal volume of patients from several countries is given. In descendant order, the patients with the bigger hippocampal volume are from Turkey (Özdemir et al., 2019), Italy, the United States (De Francesco et al., 2021), Norway (Ystad et al., 2009), China, Austria (Mangesius et al., 2022) and Ecuador, followed by those that present the smaller hippocampal volume such as The United Kingdom (Cook et al., 1992) and Cuba (Viña-González et al., 2021). Hence, note that Ecuador (Quito) has patients with small hippocampal volumes in relation to Mediterranean and northern hemisphere countries (such as the United States and Norway). Moreover, the hippocampi of patients in countries that are islands (Cuba and the United Kingdom) report smaller volumes than those from Ecuadorian patients.

Now, the results from a statistical analysis to determine if the data of participants from Quito is significantly different from the data of other countries are described. The normality of the data is determined by applying the Kolmogorov–Smirnov test, finding that the data tends to be normally distributed (*p =* 0.75). Considering that the data from the other studies is normally distributed as well, the hippocampal volume of Ecuadorian patients is compared to the other studies using a two-tailed *t*-test (*p* < 0.05), summarizing the results in Table 1. It is worth noticing that most of the hippocampal volumes from other countries are significantly different to those from Ecuadorian patients, except for the hippocampal volumes of Cubans, and from healthy, as well citizens with Temporal Lobe Epilepsy (TLE) from The United Kingdom, where a significant difference among the corresponding data was not detected.

#### 3.1.2 Classification by sex

**Figure 7:**
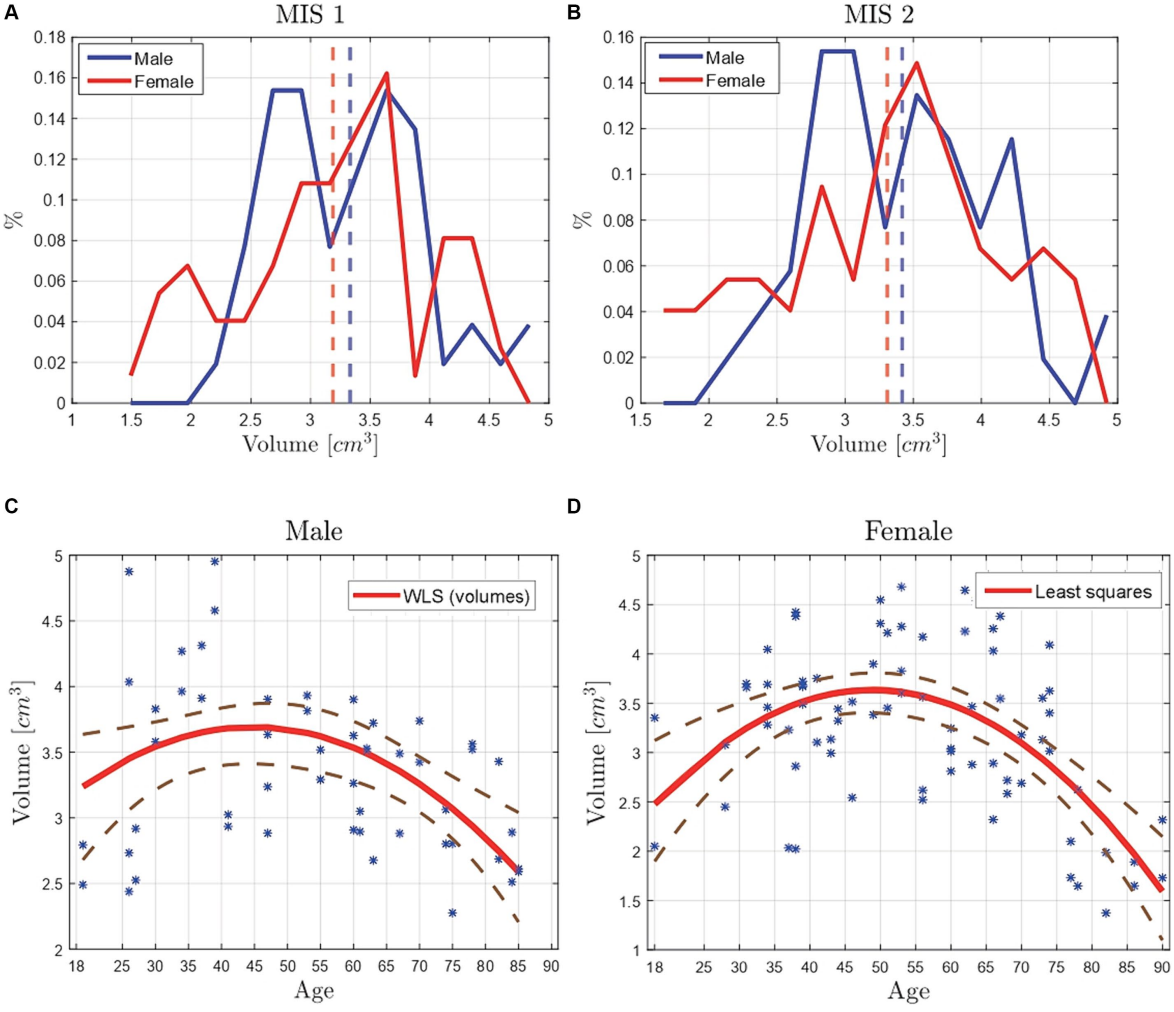
Volume comparison of the manually segmented hippocampi of 26 male and 37 female Ecuadorian patients by two MIS. The vertical dotted lines represent the mean values of the data. **(A)** Volume distribution from the MIS 1 data, yielding a difference of 0.14 cm^3^ between male and female’s mean values. **(B)** Volume distribution from the MIS 2 data, yielding a difference of 0.14 cm^3^ between male and female’s mean values. **(C)** Scatter plot of the hippocampal volume of 26 male Ecuadorian patient’s vs. age, fitting the data by means of the WLS method. **(D)** Scatter plot of the hippocampal volume of 37 female Ecuadorian patients vs. their age, fitting the data by means of the least squares method.

In Figures 7A,B the hippocampal volume distribution after classifying them by their patience’s sex is shown. Studies such as (Yagi and Galea, 2018) mention that even if men typically have larger hippocampal volume than women, no statistically significant sex differences are reported. In the present work, it is observed that the mean volume of male and female patients is similar (see Figures 7A,B). Hence, the MIS 1 data yields a mean value of 3.33 cm^3^ for males and 3.19 cm^3^ for females, giving a difference of 0.14 cm^3^. Furthermore, the MIS 2 data yields a mean value of 3.42 cm^3^ for males and 3.31 cm^3^ for females, representing a difference of 0.11 cm^3^.

The results suggest that, in average, male’s hippocampi are larger than women’s. However, after comparing the hippocampal volumes classified by sex with a two tailed *t*-test, no statistically significant difference was found (*p =* 0.27). This is consistent with the study by (Yagi and Galea, 2018), but differing from the results reported in the study from Turkey (Özdemir et al., 2019).

To analyze the behavior of the hippocampal volume by sex, and with respect to age, two additional scatter plots are included. One with the data points of 26 male patients, and another with 37 female patients, as depicted by Figures 7C,D.

In Figure 7C, the data is fitted with the WLS method, classifying the hippocampal volumes by age in 5-year intervals (starting from 18 to 95 years), using the average volume corresponding to each age range as weights, yielding a *R*^2^ of 0.28. Moreover, in Figure 7D the data is fitted by means of the Least squares method with quadratic polynomials, yielding a *R*^2^ of 0.36. It is worth noticing that the best fit (greater *R*^2^) was yielded by the scatter plot corresponding to the female patients, that is, due to more data points available to be fitted in comparison with male patients.

The behavior of both curves is similar to the plot of the whole data (see Figure 7D), were the volume increases until a certain age and then starts to decrease. In case of male patients, the curve starts at an approximate volume of 3.2 cm^3^ at 18 years old, it rises until a maximum volume of 3.7 cm^3^, reached at age 45, and then it starts to decrease until a minimum volume of 2.6 cm^3^, reached at age 85. For female patients, the curve starts at an approximate volume of 2.5 cm^3^ at 18 years old, rising until a maximum volume of 3.6 cm^3^ is reached at age 50, finally decreasing until a volume of 1.6 cm^3^, reached at age 90. Moreover, it has been reported that a modest reduction in hippocampal volume exist in male and female patients under 50 years old, but in patients over 50 years old the volume loss is of approximately 1.2% per year (Bettio et al., 2017).

Now, in order to compare the results obtained by researchers in other countries with the already available data from Ecuadorian patients (HipoML, 2023) (see Table 1), the hippocampal volumes were matched with respect to age range, as displayed by Table 1. It is worth mentioning that, from now on, the data from the Austrian study is discarded, since its study focuses on comparing the results given by different automatic segmentation methods. The study from China is also discarded since most of its data is from patients under the age of 18 years old, being this information that was not included in this study.

Table 1 suggests that the hippocampi of male patients have, in general, greater volumes than those of female patients, being consistent with the analyzed results shown by Figure 7. However, the results reported in the study from The United Kingdom suggests that the hippocampal volume of healthy female patients is greater than the hippocampal volume of their male counterparts.

#### 3.1.3 Classification and analysis by brain’s hemisphere

**Figure 8:**
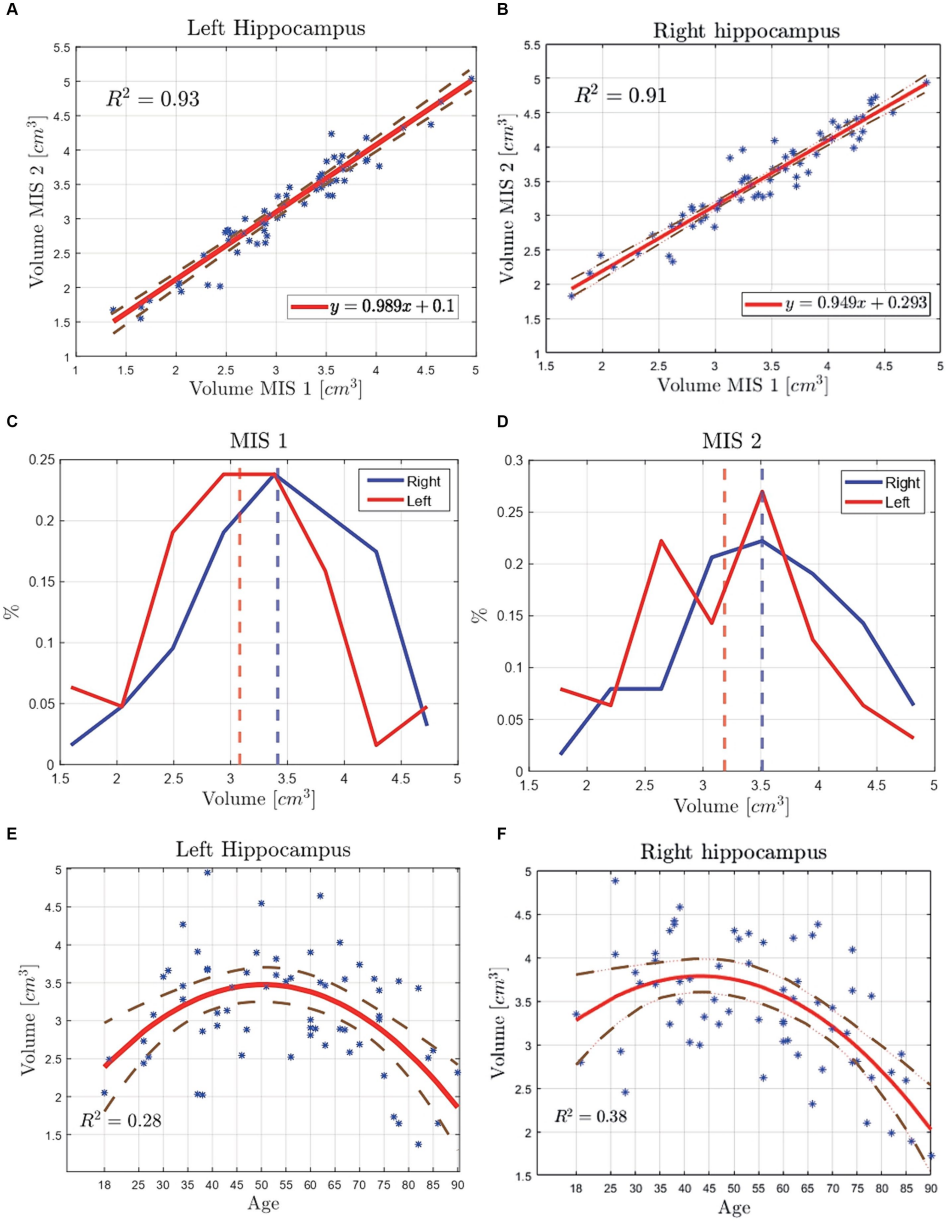
Volume comparison of the manually segmented hippocampi of 63 Ecuadorian patients by two MIS. The vertical dotted line represents the mean value of the corresponding distribution. **(A)** Scatter plot with the volume data points of the left hippocampus, reporting a *R*^2^ = 0.93. **(B)** Scatter plot with the volume data points of the right hippocampus, reporting a *R*^2^ = 0.91. **(C)** Volume distribution from MIS 1 data, yielding a difference of 0.33 cm^3^ between the right and left hippocampi’s mean values. **(D)** Volume distribution from MIS 2 data, yielding a difference of 0.32 cm^3^ between the right and left hippocampi’s mean values. **(E)** Scatter plot of the left hippocampus’ volume classified by brain’s side vs. age. The data was fitted implementing the least squares method (LSM). **(F)** Scatter plot of the right hippocampus’ volume classified by brain’s side vs. age. The data was fitted implementing the least squares method (LSM).

In this subsection, first, the hippocampal volume computed from the manual traces of the MIS is compared by the brain’s side, analysing their correlation. In Figures 8A,B a scatter plot of 63 data points, corresponding to the left and right hippocampus volume is displayed. The study of both plots suggest a strong correlation of the data, with a *R*^2^ = 0.93 and *R*^2^ = 0.91 for the left and right hippocampus, respectively. A similar study (Chaves et al., 2018) compared the results of the hippocampal volume from a manual segmentation procedure by two operators, reporting (for 28 data points, i.e., 14 patients with ages between 65 and 84 years) a correlation coefficient of 0.86 and 0.88 for the left and right hippocampus, respectively.

In Figures 8C,D the hippocampi volume distributions are displayed. The respective mean values, in Figure 8C, are of 3.08 cm^3^ for the left hippocampi and 3.41 cm^3^ for the right hippocampi, corresponding to a difference of 0.33 cm^3^. Both left and right hippocampi have a maximum volume of 4.75 cm^3^. However, the left one has smaller volumes in comparison to the right one, reporting a minimum value of 1.25 and 1.75 cm^3^, respectively. The results from Figure 8D are similar, yielding mean values of 3.19 cm^3^ for the left hippocampus, 3.51 cm^3^ for the right hippocampus, and a difference of 0.32 cm^3^. Finally, between both MIS’ data, the average right hippocampal volume is of 3.14 and 3.46 cm^3^ for the left one, suggesting that the left hippocampus is smaller in volume than the right hippocampus.

As depicted in Figures 8C,D, the right hippocampus’ volume is greater than its left counterpart. Furthermore, a statistical analysis is performed to determine if this difference is statistically significant. The Kolmogorov–Smirnov normality test determined that the data corresponding to the right (*p =* 0.99), and left hippocampus (*p =* 0.51) tend to follow a normal distribution. Thus, a two-tailed *t*-test (*p* < 0.05) is applied, finding a significant difference between the right and left hippocampus (*p =* 0.02), stating that the volume of the right hippocampus of Ecuadorian patients is statistically greater than that of the left hippocampus.

Following the same procedure as in subsubsection 3.1.2, a scatter plot (see Figures 8E,F) is used to compare the behavior of right and left hippocampal volumes throughout age. In Figures 8E,F, the approximate maximum volume 3.5 cm^3^ of the left hippocampus reached at age 50 years old, the right hippocampus reaches its maximum volume of approximately 3.7 cm^3^ at age 45 years old.

Finally, the left and right hippocampal volumes of Ecuadorian patients and participants from other studies are compared and reported by Table 1. It can be safely stated, and consistent with what has been observed in this study, that the right hippocampus has, on average, a greater volume than its counterpart, except for healthy patients and those with TLE reported in the study from the United Kingdom. Furthermore, the hippocampal volume, in descending order, shows similar results to those observed in Table 1, with the difference that the right hippocampus of patients from Bergen (Norway) has a greater volume than the patients from Italy. On the other hand, the volume of the left hippocampus, in descending order, also shows similar results to those observed in Table 1, with a difference when it comes to the healthy patients, and patients with TLE from the United Kingdom, which have a greater volume than Ecuadorian patients.

### 3.2 Other morphological parameters (data analysis and correlation)

**Figure 9:**
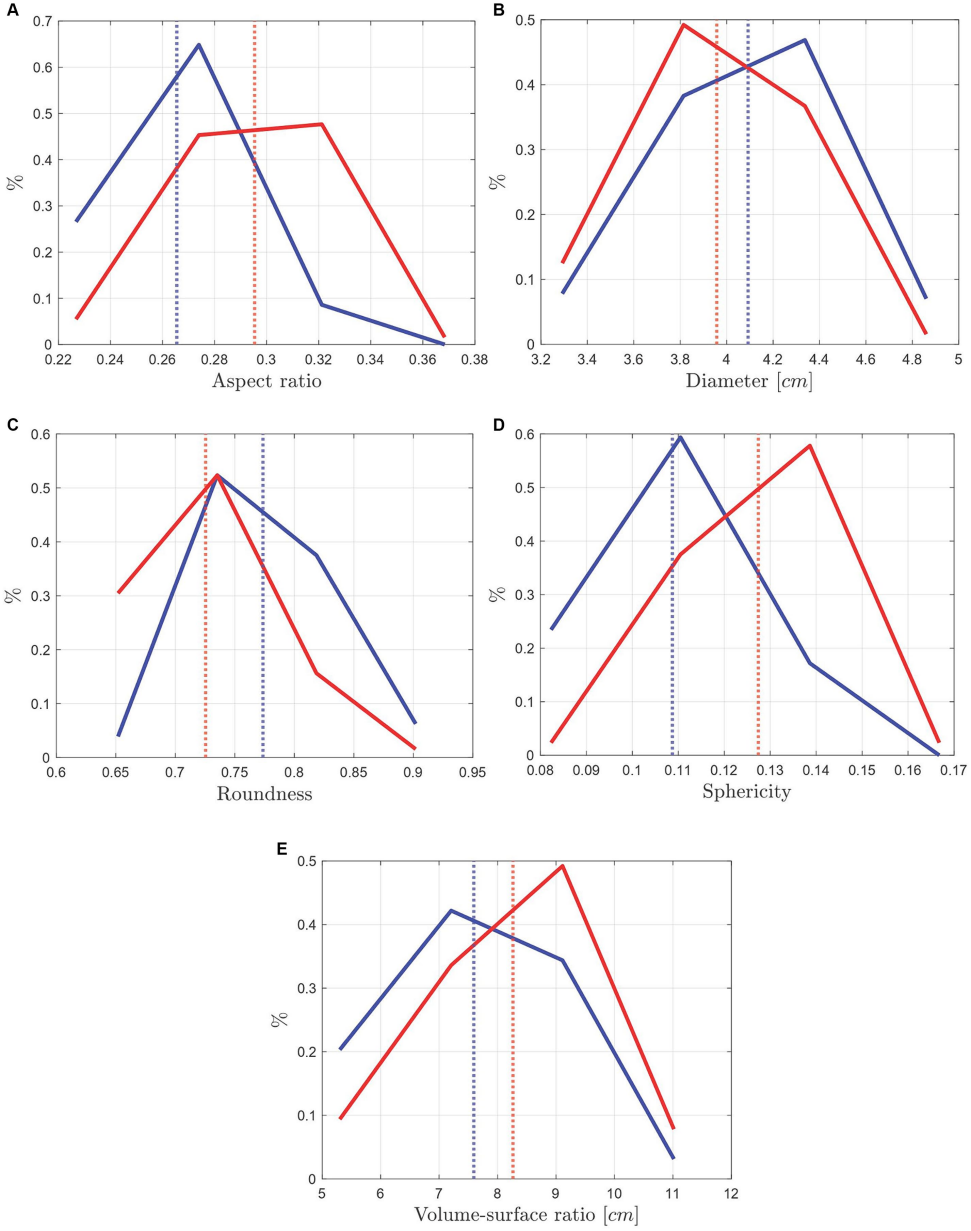
Morphological distribution comparison of the manually segmented hippocampus of 63 Ecuadorian patients by two MIS. The vertical lines represent the mean value of the data. **(A)** Hippocampus’ aspect ratio distribution comparison, with an approximate mismatch of 7.14% between the mean values of the two MIS. **(B)** Hippocampus’ diameter distribution with an approximate mismatch of 3.23% between the mean values of the two MIS. **(C)** Hippocampus’ roundness distribution with an approximate mismatch of 5.33% between the mean values of the two MIS. **(D)** Sphericity distribution with an approximate mismatch of 16.78% between the mean values. **(E)** Volume to surface ratio distribution with an approximate mismatch of 8.47%.

In this section, we introduce a first analysis of other morphological parameters that may be related to the hippocampus “health,” taking advantage of the level-set technology that enables us to obtain faithful 3D digital representations of the hippocampal morphology. This, in turn, allows the qualitative and quantitative computing of any morphological descriptor. In Figure 9A the aspect ratio (defined in Subsection 2.3) distribution is displayed. The distribution of the hippocampal aspect ratio from images segmented by MS1 (blue line) shows a smaller aspect ratio (0.27) in contrast from MIS 2, represented with the red line (0.29), yielding a difference of 0.02 and an approximate mismatch of 7.14%. The differences seen in Figure 9A suggests that the manual traces done by the MIS corresponding to the red line are thicker in comparison to the other.

In Figure 9B the diameter distribution is displayed. Diameter is defined by the smallest sphere that contains the hippocampus, where the sphere’s diameter matches the diameter of the hippocampus. The distribution of the hippocampal diameters from images segmented by MS1 (blue line) shows a greater diameter mean (4.09𝑐𝑚) in contrast of the red line (3.96 cm), representing a difference of 0.13 cm and an approximate mismatch of 3.23%. A larger diameter indicates that the hippocampi are longer, thus, the differences seen in Figure 9B suggests that the manual traces done by the MIS corresponding to the blue line are larger in comparison to the other.

In Figure 9C the roundness distribution is displayed. Roundness is a morphological descriptor indicating how rough or smooth the hippocampus is. Values far from 1 show greater roughness, while values close to 1 indicate that the hippocampus’ surface is smoother. The blue line shows a greater roundness mean (0.77) in contrast of the red line (0.73), representing a difference of 0.04 and an approximate mismatch of 5.33%. The data suggests that the manually traced hippocampus by the MIS corresponding to the blue line are rougher than the other. This difference in roundness could be due to several factors, including the tracing pulse of the MIS or the “pencil” used, since, in specialized software for medical images tracing like HOROS Project (2018), the thickness of the pencil can be adjusted.

In Figure 9D the sphericity distribution is displayed. Sphericity is a morphological descriptor indicating how similar the shape of the hippocampus is to a sphere (Madan, 2018). A value of 1 shows that the shape is a perfect sphere, while values far from 1 indicates that the shape of the hippocampus is more elongated and tend to look like a thread. The distribution of hippocampal sphericities from images segmented by MS2 (red line) shows a greater sphericity mean (0.13) in contrast to the distribution from MS1, blue line (0.11), representing a difference of 0.02 and an approximate mismatch of 16.78%. The sphericity distribution of both MIS is similar, agreeing with what has been reported.

In Figure 9E the hippocampal volume to surface ratio distribution is displayed. As its name suggests, volume to surface ratio is a morphological descriptor that relates shape to size, since it is defined as the surface area enclosing a given volume, and it could be an important indicator to determine on how shrunken the hippocampus is. The red line shows a greater volume to surface ratio mean (8.26 cm) in contrast of the blue line (7.59 cm), representing a difference of 0.67 and an approximate mismatch of 8.47%.

The data corresponding to volume to surface distribution of MS2 (red line) in Figure 9E has bigger mean values in contrast with the blue line (MS1), which, as it already was stated with the diameter in Figure 9B, may correspond to larger hippocampus, inferring that the manual traces of one specialist are longer than the other. Finally, a comparison of the average of all the hippocampal parameters’ data between MIS 1 and MIS 2 is performed and displayed on Table 2.

**Table 2:** Hippocampus’ morphological parameters comparison from both MIS data.

(A) All the morphological descriptors			
Parameter	MIS 1	MIS 2	Average
Volume	3.25 cm^3^	3.35 cm^3^	3.30 cm^3^
Aspect ratio	0.27	0.29	0.28
Diameter	4.09 cm	3.96 cm	4.03 cm
Roundness	0.77	0.73	0.75
Sphericity	0.11	0.13	0.12
Volume surface ratio	7.59 cm	8.26 cm	7.93 cm

(B) Data by sex									
Parameter	Male (MIS 1)	Male (MIS 2)	Mismatch	Female (MIS 1)	Female (MIS 2)	Mismatch	Average (male)	Average (female)	Difference
Volume	3.33 cm^3^	3.42 cm^3^	1.33%	3.19 cm^3^	3.31 cm^3^	3.69%	3.38 cm^3^	3.25 cm3	3.92%
Aspect ratio	0.26	0.30	14.29%	0.27	0.29	7.14%	0.28	0.28	0
Diameter	4.14 cm	4.11 cm	0.72%	4.08 cm	3.98 cm	2.48%	4.11 cm	3.98 cm	3.21%
Roundness	0.77	0.73	5.33%	0.78	0.73	6.62%	0.75	0.75	0
Sphericity	0.11	0.13	16.66%	0.11	0.13	16.66%	0.12	0.12	0
Volume surface ratio	7.6 cm	8.2 cm	7.59%	7.6	8.2	7.59%	7.9 cm	7.9 cm	0

(C) Data by brain’s hemisphere									
Parameter	Right (MIS 1)	Right (MIS 2)	Mismatch	Left (MIS 1)	Left (MIS 2)	Mismatch	Average (right)	Average (left)	Difference
Volume	3.41 cm^3^	3.51 cm3	2.89%	3.08 cm3	3.19 cm3	3.51%	3.46 cm3	3.14 cm3	9.69%
Aspect ratio	0.27	0.30	10.53%	0.26	0.28	7.40%	0.29	0.28	3.51%
Diameter	4,14 cm	4.01 cm	3.19%	4.09 cm	3.93 cm	4,00%	4.08 cm	4.05 cm	1.73%
Roundness	0.76	0.71	6.8%	0.79	0.74	6.54%	0.74	0.77	3.97%
Sphericity	0.11	0.13	16.66%	0.10	0.12	18.18%	0.12	0.11	8.69%
Volume surface ratio	7.88 cm	8.59 cm	8.62%	7.34 cm	7.95 cm	7.98%	8.24 cm	7.65 cm	5.16%

The manual segmentation process performed by both MIS was tuned and calibrated with respect to the hippocampal volume. Thus, the objective was to reduce the approximate mismatch in volume, and in the same way, to verify if the mismatch from the other morphological parameters was low. Based on the results displayed in Table 2, most of the morphological parameters’ values from both MIS are acceptable, with mismatch percentages under 10%, except for sphericity. However, the mismatch in sphericity has greater numerical sensitivity since its values are closer to zero.

When relating sphericity and aspect ratio from Table 2 (which are similar morphological parameters), it is worth noticing that the average data from MIS 2 reports greater values than MIS 1. Moreover, data from MIS 1 reports greater average diameter than MIS2. This would suggest that the hippocampi traced in the simple brain MRIs by MIS 2 are thicker and less elongated in comparison to the traces performed by the MIS 1. However, the mismatch in diameter is less than sphericity and aspect ratio, indicating that the main difference in the traces focuses on its thickness. The analysis is consistent with the mean values of volume and volume to surface ratio, which are greater in the data from MIS 2. Hence, the surface area from the traces by MIS 2 should also be greater, also suggesting that the manually traced hippocampi are thicker.

#### 3.2.1 Morphological data analysis by sex

In this section, we attempt to unravel unknown patterns among the already described morphological descriptors. First splitting the data by sex.

**Figure 10:**
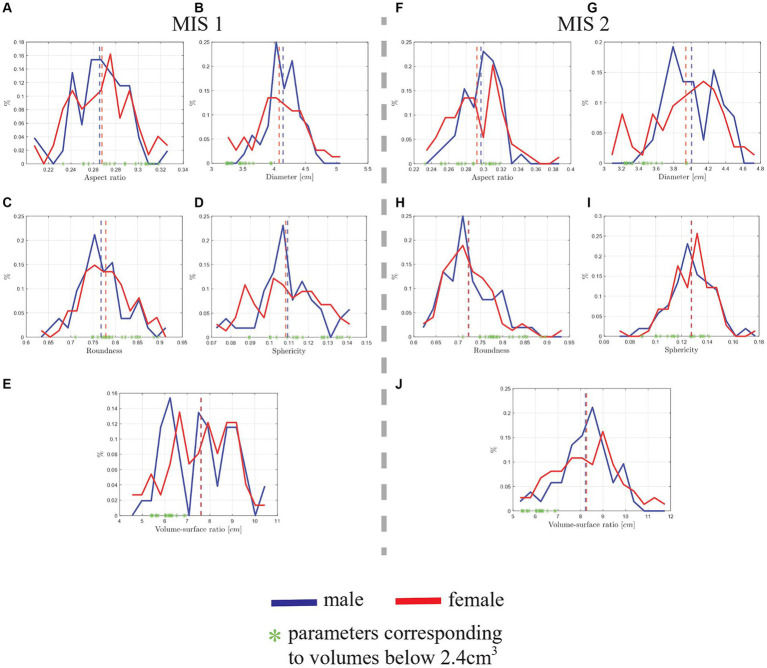
Morphological distribution comparison of the manually segmented hippocampus of 26 male and 37 female Ecuadorian patients by two MIS. The vertical dotted lines represent the mean value of the data. **(A)** Aspect ratio distribution of MIS 1, yielding a difference of 0.01 between male and female’s mean values. **(B)** Diameter distribution from MSI 1, yielding a difference of 0.06 cm between male and female’s mean values. **(C)** Roundness distribution from MSI 1, yielding a difference of 0.01 between male and female’s mean values. **(D)** Sphericity distribution from MIS 1, yielding little difference between male and female’s mean values. **(E)** Volume to surface ratio distribution of the first MIS’ data (MIS 1), yielding no difference between male and female’s mean values. **(F)** Aspect ratio distribution of MIS 2, yielding a difference of 0.01 between male and female’s mean values. **(G)** Diameter distribution from MSI 2, yielding a difference of 0.07 cm between male and female’s mean values. **(H)** Roundness distribution from MSI 2, yielding no difference between male and female’s mean values. **(I)** Sphericity distribution from MIS 2, yielding no difference between male and female’s mean values. **(J)** Volume to surface ratio distribution of the second MIS’ data (MIS 2), yielding no difference between male and female’s mean values.

It is worth pointing out that some female’s hippocampal volumes are much smaller than male’s, as previously displayed by Figures 7A,B. Hence, we were interested to analyze if the lowest values of volumes were related to other morphological descriptors. Thus, in Figure 10, the morphological parameters that correspond to hippocampal volumes that are lower or equal than 2.3 cm^3^, according to the image segmentation by MIS 1, and lower or equal than 2.4 cm^3^, according to MIS 2, are highlighted with green dots (located in the x-axis of the plot).

In Figures 10A,F, the aspect ratio (defined in Subsection 2.3) distribution by sex is displayed. From the data of MIS 1, the distribution indicates similarities in the hippocampi of male and female patients, with mean values of 0.26 and 0.27, respectively, which corresponds to a difference of 0.01 (3.77%). Thus, the aspect ratio of female’s hippocampi is slightly bigger than male’s, suggesting that are more elongated. On the other hand, the data from the second MIS suggests that male’s hippocampi are slightly more elongated than female’s, with mean values of 0.30 and 0.29, respectively, and a difference of 0.01 (3.39%). In average, the hippocampal aspect ratio of male and female patients correspond to 0.28. In terms of aspect ratio, the hippocampi of male and female patients do not seem to have a significant difference, on the contrary, they present similar distributions. Also, no relationship was found with the hippocampi that yielded the smallest volume values, since, as shown in Figures 10A,F, the aspect ratio values that correspond to the aforementioned volumes are distributed throughout the x-axis of the plots.

In Figures 10B,G, the hippocampal diameter distribution by sex is displayed. The distribution, from MIS 1, indicates similarities in the mean diameter value of the hippocampus, with values of 4.14 cm for male patients and 4.08 cm for female patients, corresponding to a difference of 0.06 cm (1.46%). Moreover, the similar data by MIS 2 yields mean values for male and female patients of 4.01 cm and 3.94 cm respectively, and a difference of 0.07 cm (1.76%). In average, the hippocampal diameter of male and female patients correspond to 4.11 and 3.98 cm, respectively. The distribution’s maximum and minimum values suggest that there are an important number of hippocampi, of female patients, that are smaller and more elongated than those of male patients. Furthermore, from the mean values, it can be inferred that the traces of MIS 1 are slightly more elongated than the performed by MIS 2. Analyzing the green points on the horizontal axis, it is worth pointing out that hippocampal volumes smaller than 2.4 cm^3^ correspond to diameter values that are located from the mean to the left (smallest values), thus suggesting that the hippocampi that are losing volume also decrease in length, which would suggest that they also reduce their elongation.

In Figures 10C,H the roundness distributions by sex are displayed. Similarly as for the diameter, from the data of MS 1, it can be inferred that both male and female patients have similar mean values in roundness, with 0.77 and 0.78, respectively, representing a difference of 0.01 (1.29%). Thus, suggesting that female’s hippocampi are a little smoother than male’s. However, the data from MSI 2 yields a different result, giving the same mean value in roundness for male and female’s hippocampi. This suggests that, on average, there is no difference in their roundness.

Now, focusing on the green dots in the plot, a pattern can be found, it is noted that the roundness values, i.e., hippocampi with volumes smaller than 2.4 cm^3^ are located from the mean to the right. This may indicate that the hippocampi with smallest volumes are also the ones with smoother surfaces. With the passing of time, the hippocampi may not only lose volume, but also their surface may become smoother, thus being related to the greater roundness values.

In Figures 10D,I the sphericity distribution by sex is displayed. The first MIS’ data yields similar mean values for male and female’s hippocampi sphericity, with 0.11 for both groups. Moreover, it is worth pointing out that the sphericity distribution from MS2 has the same mean value of 0.13. In average, the hippocampal sphericity of male and female patients correspond to 0.12. This plot suggests that the hippocampi of male and female patients do not show a significant difference in terms of sphericity. These results are contradictory to the ones seen in Figure 10A, since the distributions corresponding to MIS 1 suggest that, in average, female’s hippocampi are a little more elongated than male’s, and from the sphericity distribution it is inferred that both hippocampi are equally elongated. Furthermore, the distribution from Figure 10F suggests that, in average, male’s hippocampi are more elongated than female’s, while the sphericity distribution displays no difference in the elongation of the hippocampi. Thus, in terms of sphericty, the results are not conclusive. Also, no correlation was found with respect to the smallest hippocampal volumes, since, as can be seen in Figures 10D,I, the sphericity values that correspond to the aforementioned volumes are distributed throughout the graph without a clear pattern or showing clustering.

In Figures 10E,J the volume to surface ratio by sex is displayed. It is observed that, in Figure 10E, the mean value of 7.6 cm is the same for male and female patients. However, the distributions are slightly different, for example, the minimum volume to surface ratio for male patients corresponds to 5.5 cm, while for female patients to 4.5 cm, corresponding a difference of 1 cm. Hence, suggesting that some hippocampi of female patients are smaller than those of male patients, an observation already stated in the analysis done for Figures 7A,B (hippocampal volume distributions) and Figures 10B,G (hippocampal diameter distributions). Furthermore, similar results are displayed in Figure 10J, with a mean value of 8.2 cm.

It is inferred that volume, diameter, roundness, and volume to surface ratio are parameters related between each other, since all of them suggest the same behavior for hippocampi of male and female patients. Thus, analyzing the green points from Figure 10, the same results are observed as with diameter, where the volume to surface ratio values corresponding to the hippocampal volumes lower than 2.4 cm^3^ are located close to the mean toward the smallest values, implying that hippocampus with lower volumes could be shrunken (have a reduction of volume in relation to its surface), making its surface smoother. From the aforementioned results, the mismatch between both MIS for male and female patients is displayed in Table 2.

It is worth noticing that most of the morphological parameters’ values from both MIS are acceptable, with mismatch percentages under 10%, with the exception of aspect ratio and sphericity. As aforementioned, aspect ratio and sphericity are parameters related between each other, and are numerically sensitive since their values are closer to zero. Moreover, the analysis is similar to the one performed with the data from Table 2. Finally, a comparison between average male and female hippocampal morphological parameters is also displayed in Table 2. In average most of the morphological parameters are the same for male and female patients, except for volume and diameter, were a difference of 3.92 and 3.21%, respectively, is reported.

#### 3.2.2 Morphological data analysis by brain’s hemisphere

**Figure 11:**
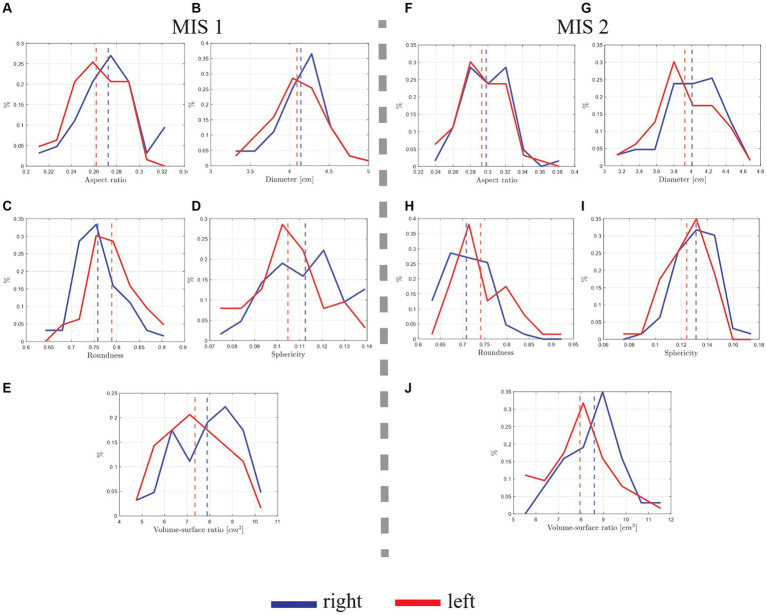
Morphological distribution comparison of the manually segmented hippocampus of 63 Ecuadorian patients by two MIS. The vertical dotted lines represent the mean value of the data. **(A)** Aspect ratio distributions from MIS 1, yielding a difference of 0.01 between the right and left hippocampi’s mean values. **(B)** Diameter distribution from MIS 1, yielding a difference of 0.05 cm between the right and left’s hippocampi mean values. **(C)** Roundness distribution from MIS 1, yielding a difference of 0.03 between the right and left hippocampi’s mean values. **(D)** Sphericity distribution from MIS 1, yielding a difference of 0.01 between right and left hippocampi’s mean values. **(E)** Volume surface ratio distribution from MIS 1, yielding a difference of 0.54 cm between the right and left hippocampi’s mean values. **(F)** Aspect ratio distributions from MIS 2, yielding a difference of 0.01 between the right and left hippocampi’s mean values. **(G)** Diameter distribution MIS 2, yielding a difference of 0.08 cm between the right and left hippocampi’s mean values. **(H)** Roundness distribution from MIS 2, yielding a difference of 0.03 between right and left hippocampi’s mean values. **(I)** Sphericity distribution MIS 2, yielding a difference of 0.01 between right and left hippocampi’s mean values. **(J)** Volume surface distribution from MIS 2, yielding a difference of 0.64 cm between the right and left hippocampi’s mean values.

In this section, we compare and contrast the morphological descriptors between the left and right hippocampus. In Figures 11A,F, the hippocampi’s aspect ratio distribution is displayed. In Figure 11A, the mean aspect ratio value of the left hippocampi is 0.26, and of the right ones is 0.27, corresponding to a difference of 0.01 (3.77%). The distribution from Figure 11B is similar, yielding 0.29 and 0.30 mean values for the left and right hippocampi, respectively, which corresponds to a difference of 0.01 (3.39%). In average, the aspect ratio of the right hippocampus is 0.29, while the left hippocampus yields an aspect ratio of 0.28. Hence, the results are consistent, suggesting that, in average, the right hippocampi are longer (more elongated) than the left hippocampi.

In Figures 11B,G the hippocampus diameter distributions are displayed. From Figure 11A, the respective mean diameter values are similar, with 4.09 cm for the left hippocampi and 4.14 cm for the right ones, representing a difference of 0.05 cm^3^ (1.22%). A similar behavior is displayed in Figure 11G, where the left and right mean values correspond to 3.93 cm and 4.01 cm, respectively, yielding a difference of 0.08 cm (2.02%). In average, the diameter of the right hippocampus is 4.08 cm, while the left hippocampus yields a diameter of 4.05 cm. Hence, the data suggests that, in average, the diameter of right hippocampi tends to be larger than the ones from the left, inferring that it may be more elongated, which was previously observed in the aspect ratio distributions from Figures 11A,F.

In Figures 11C,H the hippocampus’ roundnesses are displayed. From Figure 11C, the mean roundness value of the left hippocampi is 0.79, and of the right hippocampi is 0.76, representing a difference of 0.03 (3.87%). Moreover, from Figures 11H, a left and right mean values of 0.74 and 0.71 is reported, yielding a difference of 0.03 (4.14%). In average, the roundness of the right hippocampus is 0.74, while the left hippocampus yields a diameter of 0.77. Thus, suggesting that, in average, left hippocampi have greater roundness (their surface is smoother).

In Figures 11D,I the hippocampus’ sphericity is displayed. From Figure 11D, the mean value from the left hippocampi is 0.10, and from the right hippocampi is 0.11, representing a difference of 0.01 (9.52%). From Figures 11I, a similar behavior can be noted, with left and right mean values of 0.12 and 0.13, yielding a difference of 0.01 (8%). In average, the sphericity of the right hippocampus is 0.12, while the left hippocampus yields a sphericity of 0.11. Hence, the data suggests that, in average, the right hippocampus is slightly more elongated than the left ones, an analysis that is consistent with results from the aspect ratio (see Figures 11A,F) and diameter distributions (see Figures 11B,G).

In Figures 11E,J the hippocampus’ volume to surface ratio is displayed. From Figure 11E, the mean volume to surface ratio value of the left hippocampi is 7.34 cm, and of the right hippocampi is 7.88 cm, representing a difference of 0.54 cm (7.1%). Moreover, from Figures 11J, a left and right mean values of 7.95 cm and 8.59 cm are displayed, yielding a difference of 0.64 cm (7.74%). In average, the volume to surface ratio of the right hippocampus is 8.24 cm, while the left hippocampus yields a volume to surface ratio of 7.65 cm. Hence, the results suggests that the right hippocampi have greater values of volume to surface ratio. This may mean that right hippocampi are more shrunken in comparison to the left ones.

From the aforementioned results, the mismatch between both MIS for the right and left hippocampus, and a comparison between average right and left hippocampal morphological parameters is displayed in Table 2. It is worth noticing that most of the morphological parameters’ values from both MIS are acceptable, with mismatch percentages under 10%, with the exception of aspect ratio and sphericity.

## 4 Further improvements

The manual segmentation of the hippocampus on magnetic resonance images (MRI), carried out by medical imaging specialists (MIS), is a process that requires training time, which is also complex since it involves from reviewing each of the slices where the hippocampi are located (left and right) to performing the traces.

**Figure 12:**
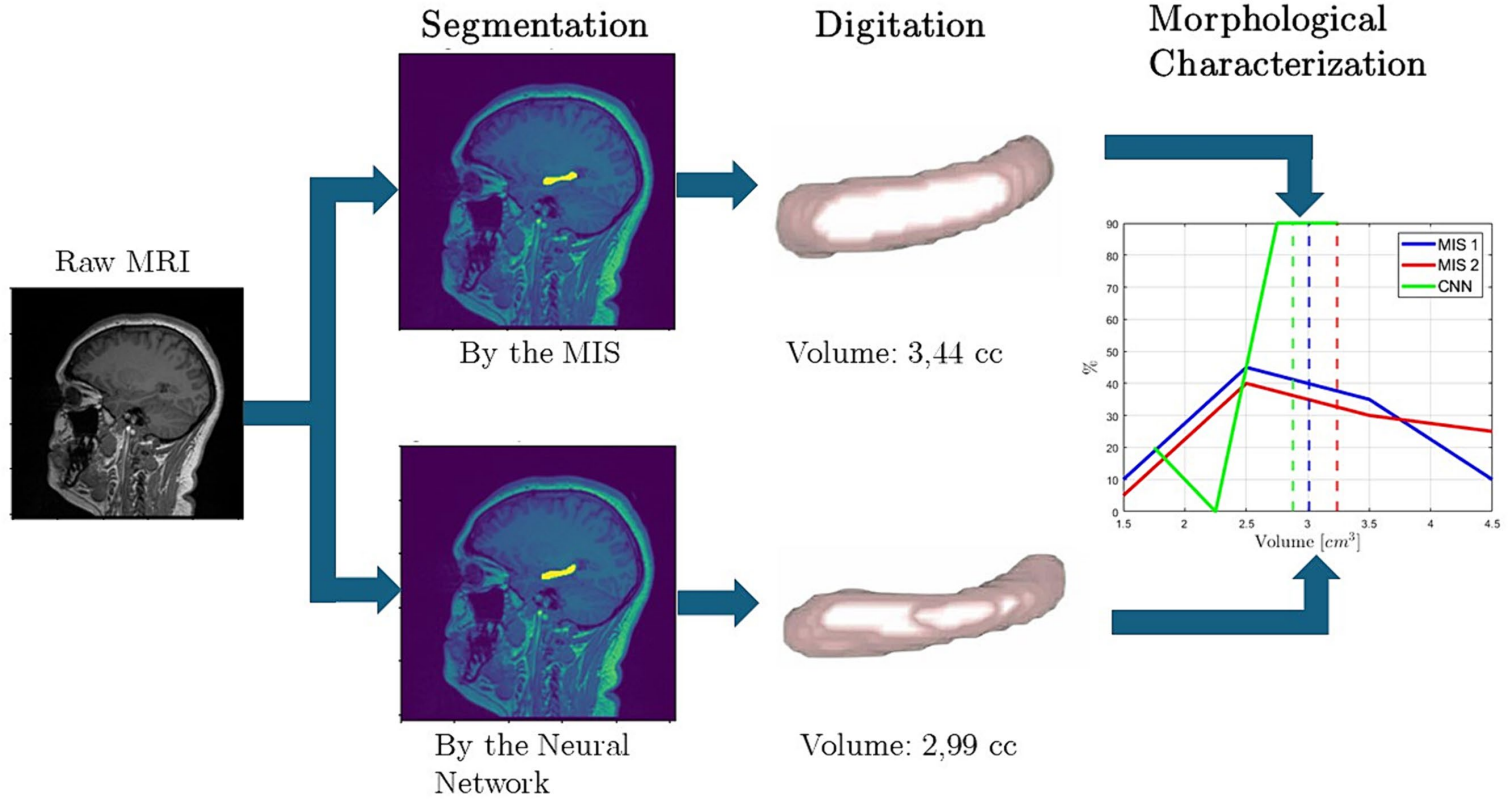
Segmentation and hippocampal morphological parameters comparison between the MIS and the trained convolutional neural network.

In an attempt to improve the hippocampus’ segmentation time, we are developing a research project where artificial intelligence’s algorithms were considered. First, the MRIs from the database HipoML were divided in training and testing datasets. Then, the images were used to train a U-net architecture (based on Convolutional Neural Networks). The results were evaluated with the Intersection over Union (IoU) metric, obtaining a score of 85%. With the current results, most of the MRIs from the testing dataset have been correctly segmented, obtaining the morphological characterization of the hippocampi and comparing them with the results from the MIS, as seen in Figure 12.

The aforementioned research is still in development, and its main conclusions and discussion will be reported in a future publication.

One of the main limitations of this study is that the MRIs from the HipoML database consist only of 63 patients from Quito. Thus, the hippocampi’s morphological characterization of people from other Ecuadorian regions are not available (which is geographically diverse). Furthermore, results can always be improved by increasing the data. Hence, for a deeper understanding, MRI of patients from all Ecuador, and also with specific mental diseases can, be considered, analyzing the morphology of their hippocampi, comparing and establishing possible biomarkers to diagnose related diseases.

## 5 Discussion

In the present work we introduce a first database with the morphological characterization of hippocampus from patients living in Quito, Ecuador. The database HipoML (HipoML, 2023) is made of raw and manually traced normal brain MRI DICOM images from 63 patients, 26 male and 37 female, aged 18–95 years old, living at an altitude above 2,500 meters, and 0.22985° of latitude. The segmented hippocampus is accurately digitized from manually traced MRIs via 3D level-set-based mathematical functions. Thus creating, for the first time, a digital twin of the hippocampus. This enables the calculation of hippocampal morphological qualitative and quantitative descriptors, such as, volume, sphericity, roundness, diameter, aspect ratio, and volume to surface ratio.

Once the hippocampal volume is obtained, it is compared and validated with respect to studies from Turkey, Italy, Unites States, Norway, China, Austria, The United Kingdom, and Cuba. No other available databases or studies from Latin American countries were found. From this analysis, it is determined that patients from Quito have an approximate hippocampal mean volume of 3.30 ± 0.74 cm^3^. Moreover, the hippocampal volume of males is greater, in average, than females, and the volume of the right hippocampus is greater, in average, than the left one. This is consistent with the results reported in the aforementioned studies, except for the one from The United Kingdom. Furthermore, the variation of volume trough age increases up to 3.7 cm^3^ for males, and 3.6 cm^3^ for females, then decreasing following an inverted U shape, being also consistent with the results introduced by the study from China (Mu et al., 2020).

Patients from Quito, Ecuador, have, in average, smaller hippocampal volumes in relation to Mediterranean and northern hemisphere countries (such as the United States and Norway). Furthermore, it is worth noticing that the hippocampi of patients in countries that are islands (Cuba and the United Kingdom) report lower average volume than Ecuadorian patients. Nevertheless, when applying a statistical test (two tailed *t*-test), no significant differences between the hippocampal volumes of Ecuadorian patients, Cubans, and the United Kingdom was found.

The difference in hippocampal volume may be caused by some variables, such as altitude, latitude, culture, diet, or climate. For instance, in this research it was reported that Ecuadorian patients have lower hippocampal volumes than patients from United States and Norway. Considering that Ecuador presents a chronic malnutrition of 17.5% on kids under 5 years old (INEC, 2023), and that the overall malnutrition rate in United States and Norway is 2.5%, one conclusion may be that diet could affect the hippocampal volume development. However, further research is needed to validate this hypothesis.

An analysis of other morphological parameters of the hippocampus is introduced for the first time, looking for correlations with the changes of hippocampus’ volume. It was found that volume, diameter, roundness and volume to surface ratio are related between each other, i.e., for smaller volumes, the diameter and volume to surface ratio also decreases, suggesting that these parameters may be indicators of how shrunken is the hippocampus. Moreover, roundness increases, which is a parameter that describes how smooth is the surface of the hippocampus, suggesting that smaller or shrunken hippocampi may have smoother surfaces. However, further research is needed.

The analysis of the morphological parameters also helps to compare the traces performed by both MIS. The manual segmentation process was tuned in relation to the comparison of the hippocampal volumes, resulting in a mismatch of 3.03%. In most cases, the mismatch between the other morphological parameters’ data was <10%. Data from MIS 2 reported greater average values of volume, sphericity, aspect ratio, and volume to surface ratio, but less values in diameter in comparison to MIS 1 data. Hence, suggesting that the hippocampi traced by MIS 2 are thicker and less elongated that the traces from MIS 1.

Finally, we are pioneering the hippocampus morphology related research in Ecuador and Latin America, offering an open access database HipoML (HipoML, 2023), beginning with the morphological characterization of the hippocampus of patients from Quito. The database can be constantly updated with MRIs of patients from other Ecuadorian regions. Specially, since Ecuador is an ethnically and geographically diverse country, all sorts of data collection are potentially representative of Latin America. In this regard, future research may include an ample comparison of the hippocampal characteristics between mestizos living at high altitude from those living at the sea level.

Furthermore, a hippocampal morphological parameters comparison can be stablished between patients of different ethnical groups living in Ecuador, i.e., African Americans, indigenous groups from the Amazon and the Andean mountains chain, and well as mestizos living in all regions. Hence, enabling accurate analysis about the influence of environmental and cultural conditions such as climate, height, or diet on the hippocampus’ morphology and its related neurological diseases. Thus, providing new research opportunities and insights not only for scientists, but also for medical practitioners and specialists such as neurologists, psychologists, and psychiatrists.

## Data Availability

The datasets presented in this study can be found in online repositories. The names of the repository/repositories and accession number(s) can be found at: HipoML, http://www.healthml.center/en/.
